# Advances in Molecularly Imprinted Polymers as Drug Delivery Systems

**DOI:** 10.3390/molecules26123589

**Published:** 2021-06-11

**Authors:** Rui Liu, Alessandro Poma

**Affiliations:** 1 UCL School of Pharmacy, 29–39 Brunswick Square, Bloomsbury, London WC1N 1AX, UK; rui.liu@alumni.ucl.ac.uk; 2 Division of Biomaterials and Tissue Engineering, UCL Eastman Dental Institute, Royal Free Hospital, UCL Medical School, Rowland Hill Street, London NW3 2PF, UK

**Keywords:** molecularly imprinted polymers, drug delivery systems, drug delivery mechanisms

## Abstract

Despite the tremendous efforts made in the past decades, severe side/toxic effects and poor bioavailability still represent the main challenges that hinder the clinical translation of drug molecules. This has turned the attention of investigators towards drug delivery vehicles that provide a localized and controlled drug delivery. Molecularly imprinted polymers (MIPs) as novel and versatile drug delivery vehicles have been widely studied in recent years due to the advantages of selective recognition, enhanced drug loading, sustained release, and robustness in harsh conditions. This review highlights the design and development of strategies undertaken for MIPs used as drug delivery vehicles involving different drug delivery mechanisms, such as rate-programmed, stimuli-responsive and active targeting, published during the course of the past five years.

## 1. Introduction

### 1.1. Drug Delivery Systems

Certain categories of pharmaceuticals, such as peptides, gene-based drugs and small molecules with low solubility and/or lipophilicity, cannot be effectively delivered using conventional formulations, due to enzymatic degradation and/or insufficient absorption into systemic circulation because of molecular size and charge. A drug delivery system (DDS) refers to a device or a formulation that vehiculates a therapeutic agent into the body, with an adequate dosage and possibly in a targeted manner, at the desired rate and time to reduce adverse effects, improve patient compliance and extend the duration of pharmacological actions [1].

The loading capacity of different DDSs can significantly affect treatment efficiency and/or dosing frequency in various ways, mainly by a change of the pharmacokinetic and pharmacodynamic profile [2,3]. In particular, polymers as drug delivery vehicles have significantly advanced the progress of DDSs production [4,5]. For polymer-based DDSs, drug molecules are normally dispersed within a polymer matrix or behind a polymer membrane, aimed at releasing drugs at a controlled rate or under certain physiological conditions. Thus, various side/adverse effects of the drug can be reduced or even significantly overcome [6,7,8,9,10,11,12,13,14,15,16].

Nevertheless, a burst release of drug is frequently observed in conventional polymeric vehicles, thus resulting in potentially severe consequences for the patients such as toxicity issues and undesirable side effects caused by temporarily high-drug dosage [5]. To date, although improved polymeric vehicles have been developed, some key knowledge gaps in terms of toxicity, physicochemical instability and lack of stimuli-responsiveness have been limitedly addressed, thus hindering practical applications [17,18,19]. On average, only one of the 5000 polymer-based DDSs that enters preclinical studies turns into an approved dosage form after ~10 years (from the initial conception to the market approval) [20].

### 1.2. Molecular Imprinting: Advanced Synthetic Molecular Recognition

Many approaches have been investigated to overcome the limitations existing in conventional polymer vehicles [21,22,23]. Amongst these approaches, molecularly imprinted polymers (MIPs) represent promising polymeric carriers that could potentially ameliorate drug delivery due to their unique features. Molecular imprinting technology can generate polymers with the ability to recognize specific target molecules. In addition to this main molecular recognition feature [24], MIPs exhibit high stability in harsh conditions (including extreme pH, presence of organic solvents, high temperature and high pressure) [25,26]. Furthermore, they possess a number of advantages such as ease of preparation, cost-effectiveness, enhanced drug loading and even potential enantioselectivity [27,28,29]. Therefore, controlled and/or targeted drug delivery can be tailored for different routes of administration and pathological conditions.

The affinity of polymers for template molecules during polymerization was first demonstrated by the seminal work of Polyakov in 1931 [30]. However, it was not until the 1970s that the pioneering work of Wulff and Sarhan [31] and Arshady and Mosbach [32] brought MIPs back into focus. Indeed, there has been increasing interest in the rational design of polymer networks for molecular recognition in various fields, such as sensors [33,34,35], separation science [36], enzyme mimics [37], synthetic antibodies [38,39,40], drug discovery [41], and drug delivery [42].

MIPs are prepared in the presence of target molecules, called “templates”, used to generate specific and complementary binding sites within the polymer matrix. This allows MIPs to recognize the templates with antibody-like affinity and selectivity [36,43]. Generally, functional monomers, template molecules, cross-linkers, initiators and solvents are all essential components to the synthesis of MIPs [5,27]. The choice of the functional monomer(s) is driven by the template molecule, and polymeric matrices are mainly prepared by free radical polymerization [44]. Nonetheless, other polymerization techniques such as controlled radical polymerization [45] [e.g., reversible addition-fragmentation chain transfer (RAFT) and atom transfer radical polymerization (ATRP)] and ring-opening polymerization approaches [46,47] (anionic, cationic and radical ring-opening polymerization) have also been used. Despite the advantages, issues associated with industrial-scale manufacturing, such as the difficulty in automatic production, currently represent a significant obstacle to the translation of MIPs from academia to the commercial stage.

**Figure 1:**
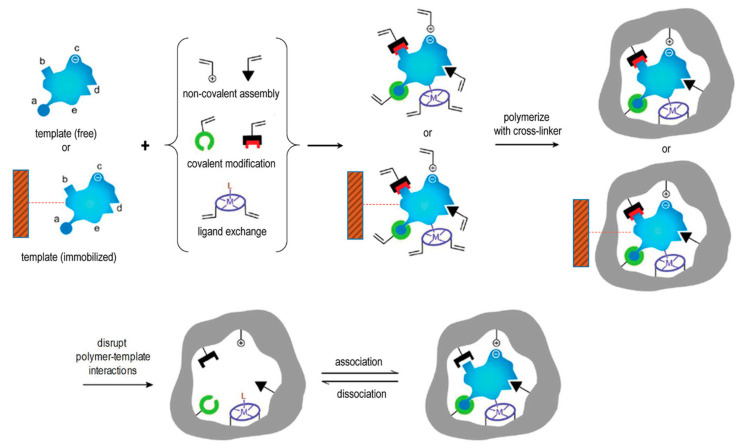
Scheme of the molecular imprinting process: the establishment of interactions between the template (free in solution or immobilized on a suitable solid support) and polymerizable groups interacting either covalently, non-covalently, or via co-ordination with a metal center with suitable functional groups or structural elements of the template. Subsequent polymerization in presence of a cross-linker develops a porous insoluble matrix containing the binding sites for the template. At this point, either the template is removed (if free), or alternatively the polymer is separated from the immobilized template in suitable washing/elution conditions. In all cases the target analyte can selectively rebind to the polymer into the sites formed by the template, or “imprints”. Reproduced with permission from Patel et al. [29].

In the conventional preparation scheme of MIPs [29], the functional molecule(s) and the free template molecule(s) (Figure 1) initially form what is known as a pre-polymerization complex, which is then polymerized in the presence of a suitable cross-linker after addition of an initiator molecule. The crosslinking monomer is crucial to stabilize this three-dimensional structure into the polymeric material. To remove the template, usually manual washings or Soxhlet extraction performed using mild acids and organic solvents are used. The template binding cavities are thus generated in the polymeric matrix, and exhibit shape, size, orientation and chemical moieties complementary to the template molecules, thereby allowing for selective rebinding.

However, the practical and commercial applications of MIPs prepared with free template molecules in solution have been limited by several issues, including template residues leftover in polymeric matrices, binding site heterogeneity and complex production processes [48]. To address these limitations, solid-phase synthesis approaches that rely on the template molecules immobilized on a solid surface have been recently developed (e.g., to prepare MIP nanoparticles, MIP NPs) (Figure 1). MIPs prepared in this fashion usually exhibit high affinity and selectivity for the target molecules and do not contain leftover template molecules, eventually leading to a final product with higher target affinity and selectivity than MIPs prepared in presence of free template molecules [49,50,51,52,53,54,55,56,57,58,59].

### 1.3. The Rationale of MIPs Used in Drug Delivery

**Figure 2:**
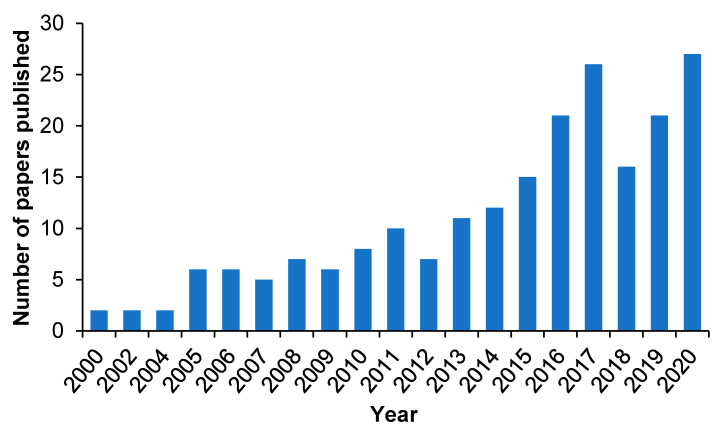
Number of published papers on MIP-based DDSs in the years 2000–2020. Source: PubMed.

The use of molecular imprinting technology in DDSs is a novel and attractive field of research, which can generate efficient polymeric DDSs with the high specificity to recognize target biomolecules and the ability to introduce stimuli-responsiveness [60,61,62]. Although MIPs-based DDSs have not been translated to the clinic yet, the molecular imprinting technology has a great potential to create ideal dosage forms. A good proof is that publications devoted to the use of MIPs in the design of novel DDSs and devices are gradually increasing [63,64,65]. Although the number of studies on drug delivery related to molecular imprinting is still limited, the studies on MIP-based drug delivery have increased significantly in the past five years, with more than 50% of the papers on drug delivery related to molecular imprinting published on PubMed during this time span, which indicates the increasing interest in the use of molecular imprinting vehicles as DDSs (Figure 2).

Additionally, owing to the high stability and robustness of MIPs in harsh conditions, MIPs have shown fascinating potential in drug delivery. Indeed, MIPs can be stored in a dry state at room temperature without losing their recognition properties for several years [5]. Furthermore, the high stability and robustness render MIPs promising vehicles for drug delivery because the imprinted cavities can protect drug molecules from complex human body environments such as within the gastrointestinal tract [66].

The considerations for the safety and biocompatibility of MIP-based DDS are extremely important. Acrylic and methacrylic monomers have been widely used in various MIP applications, and they can also be used to develop DDSs because of their biocompatibility [67]. However, the long-term toxicity of these materials when introduced in the human body needs to be further investigated. To avoid the risk of long-term toxicity, there is no doubt that the use of natural biodegradable polymers can be considered in the development of MIP-based DDS. Besides, it is also important to consider the water-compatibility of MIPs, because drug delivery vehicles usually need to be able to work in an aqueous medium to ensure efficient drug release in the body.

Norell, Andersson and Nicholls firstly reported in 1998 that MIPs can be potentially used as sustained drug delivery vehicles to release theophylline [68]. They showed that the synthesized MIPs had a higher binding affinity to theophylline than towards caffeine and demonstrated a more sustained release than the corresponding non-imprinted polymers (NIPs).

The MIP matrices rely on a high degree of cross-linking to maintain complementary cavities that act as drug reservoirs [69], but the mobility of polymer chains is often limited due to the cross-linking process [60]. Therefore, MIPs with a lower degree of cross-linking are usually more advantageous for applications in drug delivery. Furthermore, the introduction of external materials such as thermosensitive polymers [70], magnetite [71] and specific moieties such as disulphide bonds [72] allows to MIPs to exhibit suitable changes in response to external stimuli, such as changes in pressure, pH, temperature, ionic strength, electric fields, chemicals and light.

Taking into account the above-mentioned aspects, as well as the exponential increase in literature examples of MIP-based DDSs during the course of the past five years, this review aims to highlight the design and development of strategies undertaken for MIPs used as drug delivery vehicles involving three main drug-delivery mechanisms: (i) rate-programmed drug delivery, where MIPs serve as excipients to control drug diffusion and/or enhance loading capacity for the system; (ii) stimuli-responsive drug delivery, in which some physical, chemical and/or biological factors can activate drug release from the system; (iii) active targeting drug delivery, where MIPs act as target delivery vehicles that provide spatial control of drug release to specific sites of the body. We will strive to provide comparisons with conventional and possibly commercial examples of polymeric DDSs, bearing in mind the administration routes, to identify the main challenges that need to be addressed in order to eventually actualize the commercial translation of MIP-based DDSs. 

## 2. MIP-Based Drug Delivery Systems

### 2.1. Rate-Programmed Drug Delivery

Within the context of this release mechanism, MIPs are used as excipients to control drug release at a specific rate and/or time. Since Norell, Andersson and Nicholls first reported that MIPs could potentially serve as sustained drug delivery vehicles for theophylline release [68], it has been suggested that MIPs can be used as excipients to improve the precision of drug release, thereby reducing side/adverse effects and/or improving bioavailability.

#### 2.1.1. Transdermal Route of Administration

MIP-based DDSs have been exploited for transdermal formulations due to their advantages of controlled release, enhanced drug loading and/or enantioselectivity, thereby reducing the required dosing frequency, decreasing side effects, and improving therapeutic effects [73]. Nicotine is a suitable drug candidate for transdermal drug delivery because it is highly lipophilic and thus penetrates the skin readily [74]. The underlying hypothesis is that smoking abstinence can be promoted by a transdermal formulation that releases nicotine into the skin at a continuous rate, thereby maintaining a low plasma drug concentration. However, nicotine can easily volatilize from the reservoir, thus limiting its development for transdermal formulations [44,74,75].

Ruela’s group addressed this drawback by designing nicotine-based MIP particles that were dispersed in mineral oil (a non-polar vehicle) to prepare a transdermal formulation [44]. The loaded imprinted particles had high thermal and chemical stability after loading nicotine. The maximum absorption capacity of the MIP particles and the corresponding NIP particles were ~60 and ~38 µg mg^−1^, respectively, thus highlighting the effect of the presence of the selective cavities into the MIP. Skin penetration studies (on a porcine model) demonstrated that the permeation rate of the MIP formulation was close to the commercial nicotine patch Nicopatch^®^ and followed zero-order kinetics up to 48 h. The MIP formulation exhibited a longer duration of action than the commercial nicotine patches Nicopatch^®^ and Nicorette^®^ [76] (Table 1).

Although both MIPs and NIPs controlled the rate of nicotine release and penetration, MIPs could enhance drug loading and provide sustained release, thus making them more suitable as a transdermal DDS in comparison to conventional polymers [75].

#### 2.1.2. Ocular Route of Administration

The complex ocular anatomy and resistance to exogenous substances render ocular drug delivery extremely challenging. Although conventional eye drops account for 90% of all ocular medications, they achieve poor bioavailability (1–5% delivered to ocular tissue) because of pre-corneal loss [77]. Thus, frequent administrations and/or high drug concentrations are required to reach the therapeutic dose, often resulting in poor patient compliance. To overcome these limitations, ophthalmic drugs are being increasingly delivered via therapeutic contact lenses, which offer the benefits of extended wear time and highly hydrophilic matrices [78].

In recent decades, extensive research has investigated polymer-based contact lenses for ocular drug delivery, including modified hydroxyethyl methacrylate (HEMA) [79,80], silicon-containing hydrogels [81] and poly(vinyl alcohol) (PVA) hydrogels [82].

Conventional polymer contact lenses are generally considered to have low affinity for most ophthalmic drugs, so these lenses offer only a single-use formulation that delivers drugs for a few hours [83,84,85,86,87]. By contrast, MIP-based contact lenses may significantly increase drug loading and sustain drug release because of the high affinity between target drug molecules and tailored imprinted cavities.

For example, timolol is a beta-adrenergic blocker used to decrease intraocular pressure in patients with chronic open-angle glaucoma [88]. Generally, patients benefit most from local, small frequent doses because the excessive timolol release can result in ineffective absorption, thus causing side effects and requiring more frequent administration [84,89].

**Figure 3:**
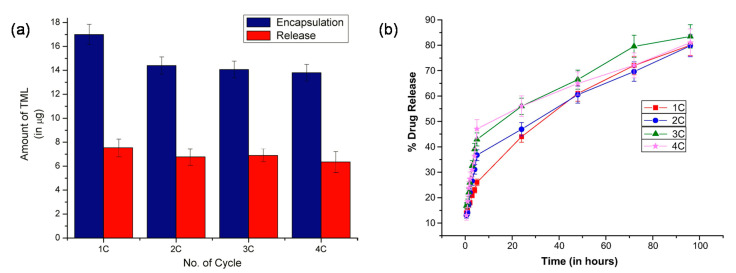
(**a**) Amount of timolol loaded and released after 24 h after the 1st, 2nd, 3rd and 4th cycles of drug loading. (**b**) Drug release profile after the 1st, 2nd, 3rd and 4th cycles of drug loading. Adapted with permission from Anirudhan et al. [89].

Anirudhan et al. studied MIP-based contact lenses that can be reusable and sustain the release of timolol [89]. Chitosan (CS) was used to improve the oxygen permeability and biocompatibility of the MIP. The MIP-based contact lenses exhibited excellent optical properties with an average visible light transmittance of ~90%. Even after multiple cycles of re-loading, the MIP-based contact lenses had a higher Young’s modulus value (0.17 MPa) than the analogous NIP-based lenses (0.06 MPa), indicating their superior strength. The drug loading was >70% over 4 drug loading cycles, respectively, and the MIP-based contact lenses achieved a sustained release of ~80% of the loaded timolol in the lacrimal fluid at 37 °C over the course of 100 h (Figure 3).

The rate and amount of timolol released were fairly stable across cycles; the amount decreased slightly for each successive cycle, but the MIP-based contact lenses were nonetheless more effective than commercial eye drops, with a release of more than 6 μg per day versus 1.70 μg per day, and more effective than conventional polymeric electrospun-coated contact lenses [90], which exhibited a much faster and uncontrolled release rate (~80% timolol released within 24 h). Although MIP-based contact lenses are thicker than normal contact lenses (900 μm versus 70–350 μm), they offer significant benefits over the conventional polymer contact lenses as a reusable vehicle for ocular drug delivery.

**Figure 4:**
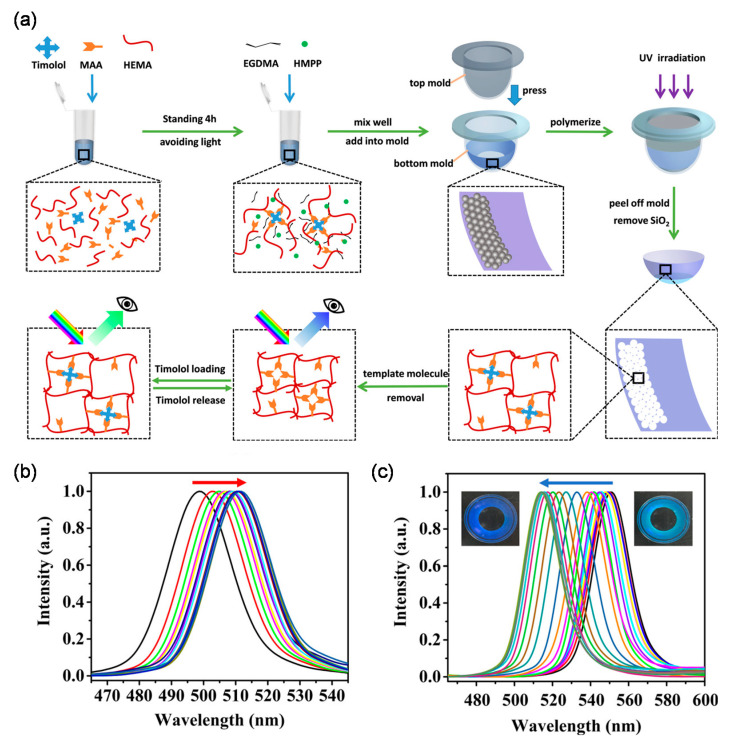
(**a**) Schematic of the preparation procedure of molecular imprinted structural color contact lenses; (**b**) change of reflection peak of the lenses during drug loading in pH 6.5 timolol loading solution; (**c**) change of the reflection peak of the lenses during drug release in ATF. Inset: the digital photographs of the lenses at the beginning of release (the equilibrium of pH, right) and at equilibrium of release (left). Adapted with permission from Deng et al. [91].

Deng et al. proposed an interesting study using reusable MIP color contact lenses, in which the extent of timolol release was quantified by the amount of visible color change in the contact lenses (Figure 4) [91].

In this system, the color change was achieved with an inverse opal architecture obtained by silicon nanoparticles (SiNPs). Owing to the presence of numerous imprinted cavities, the MIP-based contact lenses had ~2-fold higher loading capacity at pH 6.5 than the corresponding NIP-based contact lenses. In simulated lacrimal fluid (pH 8.0), timolol release was almost complete in 12 h, and no burst release was observed. It is worth mentioning that the lenses did not have any structural color in the central area of the pupil so as not to interfere with vision. Besides, the study found that the contact lenses were reusable for five cycles without a change in performance, thus indicating the potential of MIPs in the creation of a reusable and self-monitoring therapeutic contact lenses.

The hydrophilicity of polymeric matrices is usually counterproductive for the delivery of drugs that are lipophilic and/or have high molecular weight. For instance, polymyxin B is an antimicrobial lipopeptide that can be used alone or in combination with trimethoprim in commercial eye drops [92] to treat multidrug-resistant gram-negative bacteria [93,94]. However, the hydrophilicity of contact lenses is relatively incompatible with the lipophilicity and high molecular weight of polymyxin B. Malakooti et al. first reported on imprinted contact lenses that could deliver therapeutic doses of polymyxin B in 2015 [95]. The proposed contact lenses were slightly thicker (400 μm) than common contact lenses, but the oxygen permeability was similar. The lenses used the functional monomer acrylic acid (AAc) to load polymyxin B; the loading capacity of the polymers without AAc or templates (0 mg g^−1^ and 60 mg g^−1^, respectively) was lower than that of the optimal MIP formulation (100 mg g^−1^). However, the lipophilicity of polymyxin B impeded light transmittance: the MIP formulation transmitted less than 45% of light when loaded with more than 25 mg of polymyxin B. This limitation may impede the development of MIP-based contact lenses for loading lipophilic drugs, but the in vitro release study performed in saline solution showed that the MIPs sustained drug release for up to 14 days.

Importantly, MIPs can enhance the load and duration of release of lipophilic and/or high-molecular-weight drugs compared to the low affinity of conventional, non-imprinted polymers. Although some MIP-based contact lenses are thicker than commercial contact lenses to achieve the therapeutic dose of the loaded drugs, the above cases of MIP-based contact lenses indicate their potential as potentially reusable vehicles for ocular drug delivery.

Even though conventional polymer-based contact lenses may exhibit high bioavailability, their life span is limited [96,97]. By contrast, the characteristics of MIPs make reusable contact lenses possible, and this can reduce treatment costs. Furthermore, it is possible to select and modify strategic monomers to optimize the performance of MIP-based contact lenses, especially with regards to visible light transmittance and oxygen permeability.

#### 2.1.3. Oral Route of Administration

Biologics and anti-tumor agents typically require regular injections/infusions to maintain the desired therapeutic effects [98,99], and the oral route usually achieves better compliance than parenteral administration [99]. Nevertheless, oral administration for these drugs is frequently impeded by physicochemical instability, physiological barriers (such as the extreme pH in the stomach), enzyme degradation in the intestine and poor intestinal permeability [98].

**Figure 5:**
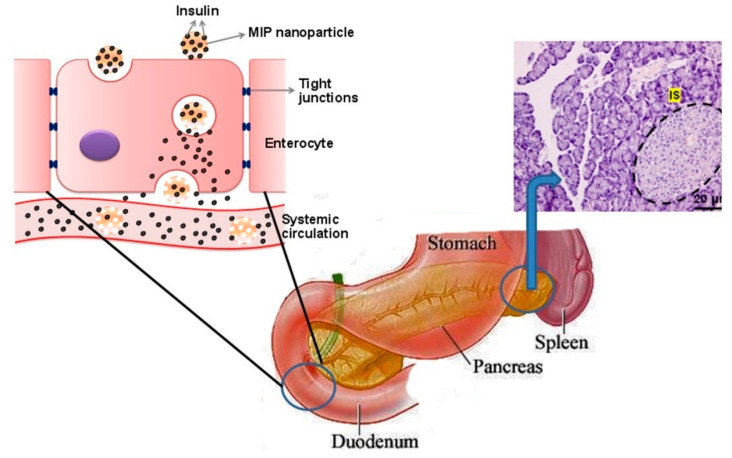
Schematic of the transport of insulin-loaded MIP NPs across the intestinal epithelial cells through the oral route and of the insulin release by endocytosis and transcytosis through the enterocytes, supplementing the lack of function of pancreatic beta cells. Reproduced with permission from Paul et al. [100].

Paul et al. proposed a molecularly imprinted oral insulin DDS to evaluate the possibility of delivering therapeutic doses of insulin via the oral route [100] (Figure 5). Insulin adsorption was higher for the MIP than the NIP, as evaluated via the adsorption of fluorescently-labelled peptide. After oral administration of the insulin-loaded MIP and NIP NPs, the results showed that the intestinal fluorescence intensity of diabetic rats treated with the MIP was nearly 10 times higher than that of rats treated with the NIP, indicating that the MIP NPs enabled more effective intestinal absorption of the drug. Moreover, at the higher pH of the gastrointestinal tract, the MIP NPs can deliver more insulin through the oral route in comparison to the NIP NPs and free insulin. After 14 days, the study found no side effects in treated diabetic rats, confirming lack of in vivo toxicity of the MIP NPs.

In another study, Paul et al. showed that the insulin loading capacity of the MIP NPs (~82%) was nearly three times higher than that of the corresponding NIP NPs [66]. The in vivo release study showed that the blood glucose level (BGL) was sharply decreased (60% within 2–4 h) after a subcutaneous injection of free insulin at a dose of 1 IU kg^−1^, whereas the BGL did not change after the oral administration of free insulin, insulin-loaded NIP NPs or blank MIP NPs.

However, the insulin-loaded MIP NPs gradually reduced the initial BGL (~40% within 4 h) at a dose of 50 IU kg^−1^ and then maintained this level for up to 12 h. Although the relative pharmacological bioavailability of the insulin-loaded MIP NPs (~2%) was lower than that of subcutaneous injections of free insulin (100%), the enhanced loading capacity and sustained release of the MIP NPs made it possible to deliver therapeutic doses of insulin. This suggests the potential to avoid multiple daily injections of insulin—a regimen that often hinders compliance due to the associated inconvenience and discomfort.

In comparison to the MIP-based DDSs, conventional polymers have also been extensively studied for oral insulin delivery (Table 2). However, insulin in these vehicles may exhibit lower loading capacity and/or higher costs. For instance, CS-gold NPs [101] were proposed for oral insulin delivery, but insulin was easily degraded by proteolytic enzymes before reaching systemic circulation. Similarly, Lopes et al. prepared CS/albumin-coated dextran (DEX) sulphate/alginate NPs [102], but no in vivo studies were performed. Moreover, gold is cost-prohibitive. Alibolandi et al. studied an amphiphilic DEX-*b*-poly(lactide-*co*-glycolic acid) (PLGA) polymersome [103]. The bioavailability of the optimal DEX-PLGA formulation with the mass ratio of insulin to polymer of 1:4 (10% at a dose of 100 IU kg^−1^) was higher than that of the MIP NPs prepared by Paul et al. (2% at a dose of 50 IU kg^−1^), but its loading capacity (25%) was significantly lower.

Capecitabin (CAP) is a commercial prodrug of 5-fluorouracil (5-FU), which can be administrated orally to treat various cancers such as colorectal cancer and metastatic breast cancer [104,105]. However, conventional CAP tablets are quickly absorbed and then converted to 5-FU, which is cleared rapidly in vivo, resulting in burst release and limited bioavailability [105]. Despite several issues, such as bioavailability, have been improved by nanoparticle-embedded microcapsules, the high toxicity caused by burst release has not been addressed adequately [104,105].

**Figure 6:**
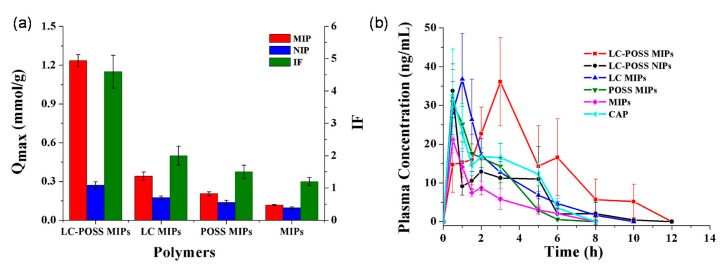
(**a**) Effects of POSS and LC on the absorptive capacity; (**b**) Plasma concentration-time curves of the loaded LC-POSS MIPs, LC-POSS NIPs, LC MIPs, POSS MIPs, MIPs and the commercial tablet of CAP. Each data point represents the mean ± standard deviation (n = 3). Adapted with permission from Mo et al. [104].

Mo et al. studied a MIP-based floating DDS that sustains the release of CAP in the stomach, thereby reducing toxic side effects and improving bioavailability [104]. The DDS used polyhedral oligomeric silsesquioxanes (POSS) to suppress non-selective sites and thereby increase drug selectivity [104], and the authors also exploited 4-methyl phenyl dicyclohexyl ethylene [a liquid crystal (LC); a physical crossing monomer with mesogenic groups] to improve the loading capacity of the MIP [106]. The LC-POSS MIP exhibited the highest loading capacity (164 mg g^−1^) in comparison to other MIPs and their corresponding NIPs (Figure 6).

In addition, the LC-POSS MIP remained buoyant for at least 24 h in water, indicating the possibility of developing a gastric-retentive DDS. The in vivo study showed that the LC-POSS MIP prolonged the time (2 h) to reach the maximum plasma concentration compared with other formulations. Besides, the CAP bioavailability was almost double than that of commercial tablets and more than 2-fold higher than that of the LC-POSS NIP and conventional MIP (Figure 6). The incorporation of LC and POSS significantly improved the performance of MIPs for the oral delivery of CAP, and the rationale behind the design of LC-POSS MIPs may provide new strategies for the development of additional sustained-release DDSs.

Conventional polymer-based oral DDSs have also been extensively studied for CAP. However, these DDSs may exhibit poor drug release behavior and/or be more expensive than MIP-based DDSs. For example, Agnihotri and Aminabhavi proposed a CS-poly(ethylene oxide-*g*-acrylamide) (PEO-*g*-AAm) hydrogel microsphere [107], which completely dissolved within 10 h. Mattos et al. developed poly(lactic acid)-*co*-poly(ethylene glycol) (PLA-PEG) nanoparticles for the oral delivery of 5-FU [108]. Nevertheless, their usage was limited by a burst release and relatively low drug loading (maximum encapsulation efficiency of ~50%). MIPs offer a cost-effective solution that also better protects drugs from complex physiological conditions.

As can be seen in the above cases, conventional polymer-based oral DDSs for biologics and anti-tumor agents have been widely studied for their potential to improve bioavailability and reduce side effects. While the encapsulation of drugs in these polymeric vehicles may prevent the inactivation and degradation of the bioactives, the in vivo characterization and/or costs of these DDSs still limit their development. The existing in vivo studies have revealed issues with uncontrolled drug release and relatively low drug loading, meaning that large doses may be required to ensure a therapeutic effect via oral administration. Unfortunately, the high-required dosage and modified polymers may cause toxicity and exhibit prohibitive costs. Also, conventional polymer-based DDSs are more likely to cause burst release in comparison with MIP-based DDSs, in which the drug molecules are physically entrapped in the high-affinity polymer matrix.

In contrast, MIPs open innovative avenues for the oral administration of biologics and anti-tumor agents because the imprinted cavities can enhance drug loading, prevent drug leakage and improve the stability of drugs in the harsh conditions of the gastrointestinal tract.

### 2.2. Stimuli-Responsive Drug Delivery

An ideal stimuli-responsive DDS should be able to provide localized, dose-controlled and rate-controlled drug delivery through endogenous stimuli (e.g., reactive oxygen species, changes in physiological pH and temperature, overexpressed proteins and enzyme levels) or exogenous stimuli (e.g., changes in temperature, light, magnetic field, etc.) [67].

Indeed, endogenous- and exogenous-responsive polymer-based DDSs have been developed for the controlled release of various drugs, especially anti-tumor agents. Nevertheless, conventional polymer-based DDSs have limitedly addressed the need for cost-effectiveness, low systemic toxicity and controlled release of drugs [109,110].

#### 2.2.1. MIP-Based DDSs That Respond to Endogenous Stimuli

Of all possible internal stimuli, changes in pH have been widely used to develop stimuli-responsive DDSs for cancer treatment because the physiological pH value of 7.4 is higher than the pH of 6 to 7 in the extracellular tumor environment [111,112]. The local environment’s reduction potential can also be a useful stimulus because most tumor cells produce a higher concentration of glutathione (GSH) than normal cells, and GSH contributes to drug resistance by eliminating reactive oxygen species [113]. Therefore, reduction-responsiveness can also be employed for stimuli-responsive MIP-based DDSs.

5-FU, a non-specific cytotoxic drug with a narrow therapeutic window [114,115], usually requires a high dose to sustain its therapeutic effects, because the drug undergoes rapid metabolism in the human body (plasma half-life of 6 min) [116]. However, the high dose poses a risk of serious side effects because of toxic metabolite accumulation [117]. Considering the advantages of MIPs, it is possible to combine molecular imprinting technology with stimuli-responsiveness to improve drug delivery.

**Figure 7:**
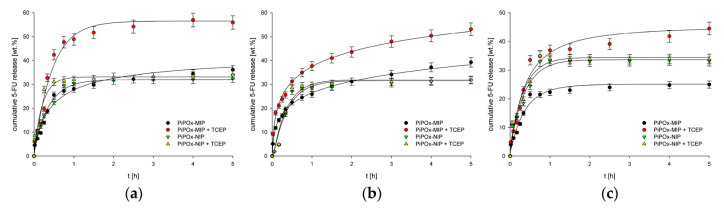
Release profiles of 5-FU from drug loaded PiPOx-MIPs and NIPs at (**a**) pH 7.4; (**b**) pH 6.5; (**c**) pH 2.0. Reproduced with permission from Ceglowski et al. [47].

Recently, Cegłowski et al. studied a reduction-responsive MIP of poly(2-isopropenyl-2-oxazoline) (PiPOx), prepared by ring-opening polymerization of iPOx, for the controlled release of 5-FU [47]. The cross-linker 3,3-dithiodipropionic acid (DTDPA) contains disulphide bonds that attract reduction-sensitive materials, thereby enabling a reduction-triggered release mechanism. The loading capacity of PiPOx-MIPs was higher than that of for PiPOx-NIPs (85 vs. 66 mg g^−1^). Furthermore, PiPOx-MIPs released ~30% of 5-FU at pH 7.4 and ~20% of 5-FU at pH 2.0 in absence of a reducing agent, whilst the drug release increased to more than 50% following the addition of tris(2-carboxyethyl)phosphine (TCEP) as reducing agent (Figure 7). Conversely, the drug was released from PiPOx-NIPs regardless from the presence of a reducing environment. This suggests that the PiPOx-MIP DDS can better control the delivery of 5-FU and lower its toxicity by improving its bioavailability in comparison with conventional polymers.

Nevertheless, it is worth noting that an initial burst release was observed for both the MIP and NIP, probably due to the aspecific surface adsorption of the drug. Moreover, an evaluation of the administration route and in vivo studies have yet to be conducted. The initial in vitro assessment, however, suggests that the PiPOx-MIPs use reduction-responsiveness to target 5-FU delivery to tumor cells more effectively than can be achieved by the conventional polymers.

Paclitaxel (PTX) is a natural chemotherapeutic agent that has been used to treat a variety of cancers, such as lung and breast cancer and acute leukaemia [45,118]. The intravenous infusion Taxol^®^ is the only approved dosage form of PTX, with severe side effects such as multi-organ toxicity frequently observed in clinical applications due to the drug’s narrow therapeutic window [45,119]. Although temperature and pH-responsive polymers were prepared for PTX delivery [119], some aspecific drug leakage was observed, which may cause toxicity.

Bai et al. proposed PTX formulated using imprinted microparticles containing methacryloyl POSS (M-POSS), which were synthesized through RAFT-controlled radical polymerization [45]. The loading capacity and encapsulation efficiency of the MIP microparticles were 1.5–3 times higher than that of the corresponding NIPs. In an in vitro release study, the release rate of PTX from the MIP microparticles was directly proportional to the M-POSS content, effect that was attributed to the increasing number of pores. Above a certain level, though, the release rate decreased as M-POSS negatively affected the formation of the imprinted cavities. Conversely, the NIP microparticles exhibited a higher release rate than the corresponding MIP microparticles, indicating that PTX was not specifically adsorbed onto the NIP. The MIP matrix could reduce PTX leakage and significantly sustain drug release. Besides, the MIP offers promising pH responsiveness that may improve the release behavior of PTX in comparison to conventional polymers and commercial Taxol^®^.

Bakhshpour et al. investigated a metal-ion mediated MIP cryogel for local administration of mitomycin C (MMC) [120], a DNA alkylating agent that can be used to treat a range of tumors. However, MMC has limited uses in clinical practice because its poor specificity causes severe acute and chronic toxicity. Motivated by this limitation, the authors prepared a MIP cryogel with high loading efficiency (70–80%) and sustained MMC release over 150 h. By contrast, the control cryogel released the same cumulative amount of MMC within 30 min. In vitro cytotoxicity studies, though, showed that the viability of non-cancerous cells in presence of the MIP cryogel was higher than 80% within 24 h, highlighting a certain level of cytotoxicity.

Implants come with a risk of postoperative infection, and the presence of antibiotics such as vancomycin (VA) on the implant surface can mitigate this risk [121,122]. However, it has been difficult to achieve long-term, controlled release of VA. For example, Yang et al. used electrochemical technology to prepare a VA-CS composite to be deposited on titanium implants [123], but 80% of the drug underwent burst release within a few hours.

Mao et al. proposed a pH-sensitive MIP NP for improving the antibacterial activity of VA [124]. The loading capacity of the MIP NPs (17%) was significantly higher than that of the corresponding NIP NPs (5%), and less than 10% of the drug was released from the MIP NPs at pH 7.4. The MIP NPs reached a steady-state concentration after 220 h at pH 5.2 and after 432 h at pH 7.4. The NIP NPs, by contrast, released all VA within 24 h at all pH values, indicating lack of pH-responsivity and inability of achieving sustained drug release. Moreover, the MIP NPs achieved an antibacterial ratio (the ratio of viable bacteria counts before and after administration) of more than 90% against *Staphylococcus aureus*, while the NIP NPs exhibited negative antibacterial ratios (i.e., *Staphylococcus aureus* actually proliferated in the presence of the implant).

From the aforementioned studies, it is clear that recent designs of MIP-based DDSs can respond to a variety of endogenous stimuli to achieve controlled and/or targeted drug release. This may be especially valuable for drugs with narrow therapeutic windows. Although additional in vivo studies are required, the existing evidence suggests that imprinting technology provides an edge to conventional polymers in terms of sustained and controlled release—an advantage that improves the therapeutic index and increases drug stability in harsh conditions. It is also worth noting that the specific composition of MIPs (particularly the cross-linker content) can markedly affect drug release behavior, and additional materials such as POSS can be used to modify MIPs to improve their performance.

#### 2.2.2. MIP-Based DDSs That Respond to Exogenous Stimuli

Exogenous stimuli, especially magnetic NPs that respond to manipulations in an external magnetic field, may also be used to trigger drug release from a MIP-based DDS. In case of the disadvantages of conventional polymers, the combination of magnetic NPs and molecular imprinting technology can be considered to generate a DDS with superior biocompatibility, stimuli-responsiveness and drug stability that reduces the severity of side effects caused by the action of the drug on non-target tissues.

**Figure 8:**
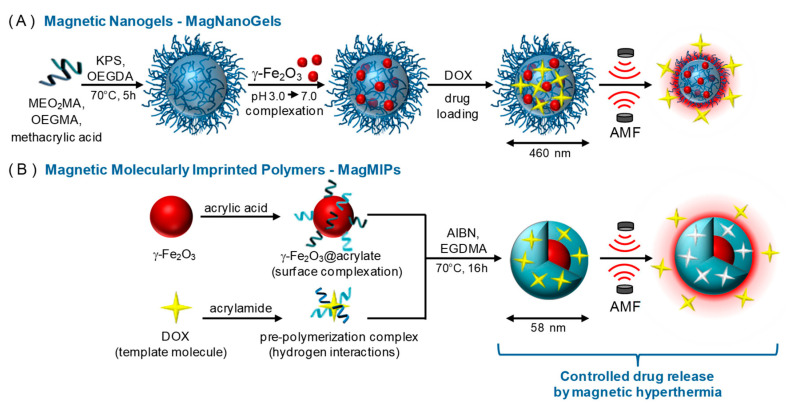
Schematic illustration of the synthesis of (**A**) MagNanoGels by precipitation radical copolymerization and post-assembly of MNPs inside nanogels and (**B**) MagMIPs via a subsequent grafting of an acrylic acid compound in the surface of MNPs and the growth of the polymer in the presence of DOX for imprinting polymerization. Loading and release of DOX under an AMF. Reproduced with permission from Cazares-Cortes et al. [71].

Cazares-Cortes et al. reported an interesting head-to-head comparison between two magnetic nanosystems for the controlled release of DOX: (i) magnetic nanogels comprised of thermosensitive polymers, and (ii) core-shell magnetic MIP NPs (MMIPs) (Figure 8) [71].

In both cases, the magnetic nanosystem is a “hot spot” that generates local hyperthermia when an external alternative magnetic field (AMF) is applied, thereby triggering the release of DOX. In in vitro drug release studies performed at 37 °C, the release of DOX from the MMIPs increased from 10% to 60% of the total payload after 4 h of exposure to the AMF via a mechanism of disrupted hydrogen bonds between DOX and the functional monomer. Similarly, the release of DOX from the magnetic nanogels increased from 24% to 45% after exposure to the AMF, but in this case, the increase in drug release was attributed to conformational changes in the gel network.

Although the MMIPs released less total DOX (7 µM) than the nanogels (16.7 µM), they released more DOX within 4 h. In addition, after exposure to the AMF, both the MMIPs and nanogels decreased cancer cell viability (from 88% to 60% and from 54% to 30%, respectively). A key limitation of the MMIPs is their relatively low loading capacity compared to that of the nanogels, meaning that larger doses of MMIPs may be required in clinical practice. However, compared to the nanogels, MMIPs offer advantages in DOX release behavior, because they achieve specific binding between the drug and the imprinted cavities while nanogels rely on physical entrapment. Therefore, MMIPs may decrease DOX leakage in systemic circulation, thereby reducing the severity of the side effects.

Another example of a DDS that responds to exogenous stimuli is the letrozole-loaded MMIP NPs (~100 nm in diameter) synthesized by Kazemi et al. for the treatment of breast cancer [125]. The in vitro release study showed that the MMIP gradually released 40% of letrozole within the first 10 h and all letrozole within 70 h, while the magnetic NIP NPs (MNIP) released more than 80% of letrozole within the first 10 h. Moreover, the drug release rate of the MMIP NPs increased with the application of an external AMF, which disrupted the weak hydrogen bonds. The release rate of the MMIP increased from 33% (no AMF) to 55% (AMF of 150 G) in 240 min at pH 7.4. Doubling the AMF strength yielded a further increase to 61% within the same timeframe. The increase in AMF strength opened the polymeric matrix further by causing the agitation and motion of the MNIP, so the MNIP and MMIP showed similar release behavior. Nevertheless, the MNIP exhibited a burst release, indicating that the MMIP may allow for better control of the drug release profile to avoid potential side effects and toxicity.

Azidothymidine is an antiviral drug historically used in the treatment of AIDS and more recently shown to have direct anti-tumor activity [126]. However, the chemical instability and short half-life (4 h) of azidothymidine in systemic circulation have limited its clinical applications [127].

**Figure 9:**
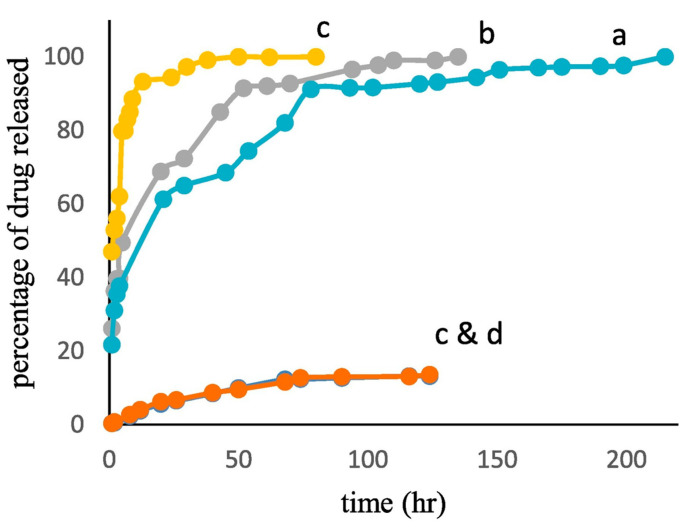
The in vitro release profile of MMIP, MIP NPs and MNIP (a, b, and c, at pH 5), MMIP and MIP NPs (c and d at pH 7.4). Reproduced with permission from Hassanpour et al. [127].

Hassanpour et al. synthesized MMIPs for the controlled release of azidothymidine [127]. The MMIPs had s significantly greater adsorption capacity (~170 mg g^−1^) than the corresponding MNIPs (~38 mg g^−1^), MIP NPs (~46 mg g^−1^) and NIP NPs (~18 mg g^−1^). In an in vitro drug release study, the MMIPs released only 14% of the loaded drug at pH 7.4 but almost all of the drug at pH 5 (Figure 9).

In addition, the MMIPs achieved ~50-fold higher cytotoxicity to MCF-7 cancer cells relative to free azidothymidine while having no effect on MCF-10 (healthy) cells. Although the effect of an AMF on the drug release behavior not assessed, the study concluded that MMIPs exhibit potential as pH-responsive vehicles to target drugs to tumors.

The combination of MMIPs and poly(*N*-isopropylacrylamide) [poly(NIPAM)] was exploited in another work for the controlled release of 5-FU. Li et al. proposed a temperature and magnetism bi-responsive MIP (TMMIP) that was obtained via surface-grafting copolymerization [70]. The TMMIP had the greatest adsorption capacity for 5-FU, followed by thymine and then uracil (~95, 84 and 81 mg g^−1^, respectively); the TMNIP had similar adsorption capacities for all three pyrimidine compounds (~60 mg g^−1^), indicating that the TMMIP (relative to the TMNIP) exhibited higher selectivity for 5-FU. The in vitro release assessed at 25 °C showed that the TMMIP released less than 70% of the loaded 5-FU within 100 min, whereas the TMNIP released 84% of the loaded 5-FU in the same timeframe. A temperature increase from 25 °C to 45 °C prompted an increase in drug release from the TMMIP (from less than 70% to about 90%), due to the thermal disruption of the imprinted cavities above the lower critical solution temperature (LCST) of poly(NIPAM).

Several examples demonstrate that MMIPs are extremely promising as drug delivery vehicles for anti-tumor agents, which are typically characterized by narrow therapeutic windows and are burdened by systemic toxicity phenomena [119]. The aforementioned studies were not limited to the effects of an AMF on drug release behavior; some MMIPs were pH-responsive instead. The influence of an AMF on TMMIPs, however, should be studied further because an AMF can generate local hyperthermia, which could result in unpredictable effects in terms of physicochemical stability as well as biological activity [128]. Nonetheless, thus far the combination of an AMF and TMMIPs enables triggered drug release without requiring external heating.

### 2.3. Active Targeting Drug Delivery

Conventional DDSs usually deliver drugs to both healthy and unhealthy tissues, even though those drugs may damage the healthy cells and thus cause severe side effects, particularly if the drugs are highly toxic (e.g., anti-tumor drugs). Active targeting is another important feature of controlled DDSs, which can spatially control the release of drugs to the target sites within the body, thereby reducing the side effects associated with damage to healthy cells, thus improving the overall therapeutic profile.

The induction of external stimuli to activate the drug delivery vehicles loaded with toxic drugs is an effective method of site-targeting as described above. If external guidance is not used, however, these DDSs will be distributed all over the body.

Therefore, polymeric DDSs with active targeting have been employed to recognize specific cell markers and deliver drugs precisely to the target sites. Active targeting of conventional polymeric DDSs is normally achieved via conjugation with the ligands of specific receptors expressed on target cells. Potential ligands include proteins, peptides, carbohydrates, nucleic acids and small molecules [129,130,131,132,133,134].

Nevertheless, actively targeted polymeric DDSs frequently face complex production processes, significantly high costs (e.g., the RGD peptide targeting sequence costs 145 USD per 10 mg) and/or poor drug release control [135,136,137,138].

In recent years, double-imprinted polymeric DDSs have been studied for active targeting drug delivery because of the unique features of MIPs as described in the introduction section.

For example, Jia et al. synthesized dual-template silicon MIP NPs used for theranostic applications towards pancreatic cancer BxPC-3 cells that overexpress human fibroblast growth-factor-inducible 14 (FN14) [139]. The 71–80 peptide of FN14 (FH) and bleomycin were used simultaneously as the template molecules to obtain the active targeting MIP NPs. Optical bioimaging technology makes it possible to use silicon NPs to diagnose cancer. The study showed that bleomycin adsorption to the MIP NPs (>4000 mg g^−1^) was 4-fold higher than to the NIP NPs (1100 mg g^−1^), suggesting that the loading capacity was markedly enhanced by the imprinting process. The FH adsorption capacity of the MIP NPs (450 mg g^−1^) was also higher than that of the NIP NPs (130 mg g^−1^), with 2.5-fold higher selectivity for the correct targeting peptide in comparison to a scrambled one. The in vitro release study performed at pH 5.3 (mimicking the tumor microenvironment) highlighted that the MIP NPs sustained the release of a total amount of 1900 mg g^−1^ of bleomycin over the course of 72 h, while NIP NPs released the drug more quickly and reached equilibrium (750 mg g^−1^) after 10 h. At pH 7.4, NIP NPs and MIP NPs released 800 mg g^−1^ and 500 mg g^−1^, respectively, after 70 h. The in vivo anti-tumor effect was evaluated in mice via a tail vein injection, and MIP NPs achieved the greatest effect. The tumor volumes of the groups treated with physiological saline, NIP NPs and free bleomycin were respectively 2.3-fold, 1.6-fold and 1.5-fold higher than the group treated with MIP NPs. These results demonstrate that MIP NPs have a superior potential for the treatment of pancreatic cancer relative to NIP NPs and free bleomycin thanks to the MIP system enhanced drug loading, specific recognition and sustained release.

**Figure 10:**
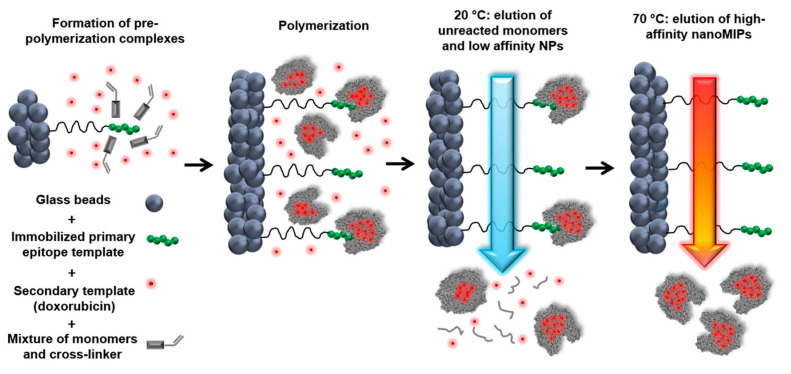
Scheme of the solid-phase synthesis process for double-imprinted nanoMIPs using a peptide epitope of EGFR as primary template attached to the solid phase and doxorubicin as secondary template in solution. Adapted with permission from Canfarotta et al. [140].

Canfarotta et al. used the solid phase synthesis approach to prepare double-imprinted MIP NPs against the epidermal growth factor receptor (EGFR) that is overexpressed in many types of tumor cells (Figure 10) [140].

An epitope of the extracellular domain of EGFR and DOX were used as the template molecules. Flow cytometry was used to analyze the selective recognition capability for MIP NPs towards breast cancer cells overexpressing EGFR. Furthermore, DOX-loaded EGFR-MIP NPs decreased the cell viability of EGFR-overexpressing MDA-MB-468 cancer cells (lower than 75%) in comparison to free DOX (more than 90%) and control NPs (~100%). Moreover, the cell viability of non-EGFR-overexpressing cell-lines was virtually unaffected, highlighting the achievement of selective targeting and DOX delivery.

**Figure 11:**
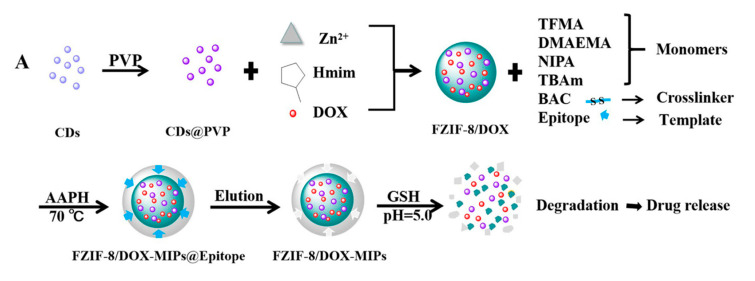

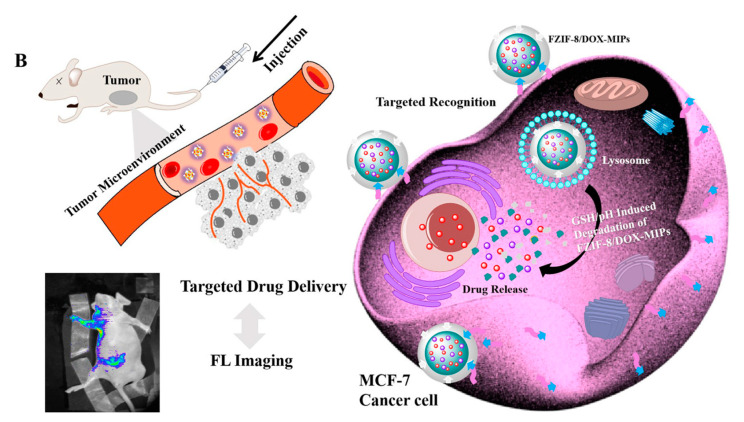
(**A**) Synthesis and GSH/pH dual stimulation degradation route of FZIF-8/DOX-MIPs; (**B**) Schematic illustration of targeted imaging and GSH/pH-responsive drug delivery of FZIF-8/DOX-MIPs. Reproduced with permission from Qin et al. [141].

Similarly, Qin et al. studied active targeting MIP NPs for the treatment of breast cancer. The MIP NPs were prepared with an epitope of the CD59 cell membrane glycoprotein and DOX as templates such that the high concentration of GSH and weak acid environment created by the cancer cells would selectively trigger DOX release (Figure 11) [141].

Fluorescent zeolitic imidazolate framework-8 (FZIF-8) was employed as the framework of the MIP NPs because FZIF-8 is fully biodegradable in an acidic environment. The fluorescence intensity of the MIP NPs was ~4-fold higher than that of the NIP NPs with 0.1 mg mL^−1^ of the epitope, indicating the enhanced adsorption and strong specificity of the MIP NPs. After 15 days and in absence of GSH, about 50% of the drug leaked from pristine FZIF-8/DOX in PBS (pH 7.4), while almost no DOX was released from FZIF-8/DOX MIP NPs in PBS (at pH 5.0 and 7.4). In the presence of GSH, the DOX release from the MIP NPs greatly increased (at pH 5.0 and 6.0) to more than 90% over 15 days. In addition, the viability of MCF-7 cells (CD59 positive) was significantly lower after treatment with the FZIF-8/DOX MIP NPs (20% cell survival after 72 h exposure to 40 μg mL^−1^) than after control treatments: FZIF-8/DOX NIP NPs (50%), FZIF-8/DOX (60%) and free DOX (60%). Importantly, normal cells did not show significant apoptosis; the anti-tumor effects were limited to the MCF-7 cancer cells. Moreover, the tumor volume (10 mm) in mice treated with FZIF-8/DOX MIP NPs was more than 2-fold smaller than that in mice treated with the controls. These results indicate that FZIF-8 MIP NPs can selectively target MCF-7 cells and trigger an adequate drug release in the presence of GSH, thereby reducing systemic toxicity and improving the therapeutic index of DOX.

As can be seen, in comparison to the conventional active targeting polymeric DDSs, double-imprinted MIP NPs offer a benefit by significantly reducing aspecific drug release and improving therapeutic effects. In addition, while biological ligands might exhibit superior specificity in certain instances, the high costs and complex production processes associated with materials such as antibodies and peptides may be prohibitive [131,142,143].

Double-imprinted polymeric DDSs, on the other hand, are extremely affordable. Solid-phase synthesis technology, in particular, makes it possible to recycle the templates depending on the production conditions, which greatly optimizes the resources whilst containing the costs [50]. Furthermore, it is facile to prepare double-imprinted polymeric DDSs in comparison to analogous conjugates [50,57,140].

## 3. Current Challenges in MIP-Based DDS

In the last 5 years, a myriad of interesting studies has revealed great potential for the development of MIP-based DDSs. Many have focused on drug delivery for anti-tumor agents, which are important candidates for improved DDSs because the majority of these drugs possess a narrow therapeutic window. Methacrylic monomers and cross-linkers such as HEMA, MAA, MBA, EGDMA and EDMA have been widely used in the advancement of MIP-based DDSs [144,145]. These monomers are considered to have acceptable toxicity and excellent biocompatibility [78,120,127], though their long-term toxicity and metabolic pathways have not been evaluated in depth. Besides, additional materials such as POSS [45,104], β-CD [45], CS [89], silicon [91,139] and stimuli-sensitive materials [47,70,124] have been used to modify MIPs to improve performance parameters such as drug release behavior, biodegradability and biocompatibility [67].

Another major obstacle in the development of most MIP-based DDSs is the use of organic solvents (e.g., toluene, chloroform) [67]. These facilitate and maintain the non-covalent interactions between the template molecules and the functional monomers [146,147]. Nonetheless, in terms of medical translation and applications, the presence of residual organic solvents can damage healthy cells, leading to serious side effects [146,148]. Furthermore, the use of organic solvents during synthesis may result in a marked difference in drug-release behavior of MIPs in aqueous media, as well as potentially increasing the manufacturing costs [148,149]. Alternatives such as supercritical carbon dioxide (scCO2) technology exhibit great environmental and safety advantages because of the unique and beneficial features of the prepared polymers, such as a controlled morphology, non-toxicity, absence of solvent residues [60] and the ability to avoid purification and drying steps [150]. Furthermore, scCO2 can stabilize hydrogen bonds between the templates and functional monomers [151]. The technology, though, is still not widely used nor easily accessible (even at a laboratory scale), possibly due to the need for specialized expensive equipment as well as the extreme operational parameters (temperatures and pressures). Nonetheless, scCO_2_ has a great potential for the development of highly pure, GMP-compliant MIP-based DDSs [150].

A further challenge that arises from the analysis of the above-discussed literature examples, is that only a limited number of studies reported in vivo data for the evaluation of MIP-based DDSs. In vivo research should be considered an indispensable step in the evaluation of any potential DDS. Since most studies evaluated toxicity and drug release only in vitro, further investigations and data are needed to ensure that the promising in vitro behavior of MIPs translates in in vivo models exhibiting safety and adequate release properties. Only through in vivo studies can we adequately evaluate the possible advantages of MIPs in clinical practice.

Another relevant concern in some cases is the inadequate drug loading of MIP-based DDSs (e.g., in contact lenses). This may result in sub-par, ineffective drug release. Importantly, though, even MIP-based DDSs with somewhat inadequate loading usually perform better than the corresponding NIP-based DDSs, indicating the potential advantages of molecular imprinting to enhance drug loading. The loading capacity may be increased by employing a smaller proportion of cross-linkers, but this strategy must also consider the trade-off between drug loading and the stability of imprinted cavities.

Furthermore, some MIPs may undergo a small initial burst release due to the adsorption of drug molecules onto their surface. For some drugs (e.g., anti-tumor agents), this may cause serious toxic effects. In other cases, however, burst release can be beneficial (e.g., at the beginning of antibiotic treatment) [42]. Perhaps better purification strategies can allow achieving an improved modulation of the burst effect. Although it is tremendously challenging to design a suitable release profile, MIPs offer ample opportunities for modifications that can implement a variety of release profiles to fit the requirements of the drug of interest.

## 4. Conclusions and Future Perspectives

Conventional drug delivery can be burdened by systemic toxicity and low bioavailability because of non-specific delivery and rapid clearance. Recently, in attempts to address these limitations, a variety of MIPs have been evaluated as novel DDSs. An analysis of the literature on MIP-based DDSs reveals a great potential for extensive research, particularly for the delivery of drugs with narrow therapeutic windows and/or low bioavailability. Although the process of designing and translating MIP-based DDSs into clinical practice is still in its infancy, ongoing development will most likely lead to the creation of innovative drug delivery vehicles with commercial value.

Imprinted polymers can not only significantly enhance the drug loading and the stability of drugs in harsh conditions but also attenuate the release behavior by engineering specific interactions between drug molecules and functional monomers. As an example of the advantages of rate-programmed drug delivery, MIPs as excipients can be used to develop sustained transdermal formulations, therapeutic contact lenses and oral formulations for protein delivery. In addition, MIP-based DDSs can be designed with stimuli-responsiveness, using properties of the imprinted cavities to achieve enhanced release profiles compared to conventional polymeric DDSs. Even more interestingly, active targeting drug delivery can be achieved via unique double-imprinting of targeting moieties and drugs.

However, much of the extant research does not go so far as to include clinical studies that address biomedical regulations regarding the novel drug delivery devices. Although some studies report the in vitro cell toxicity of MIP-based DDSs, the therapeutic effects and clinical safety cannot be determined without in vivo assessments. Therefore, additional ongoing efforts are needed to design, develop and evaluate MIP-based DDSs to evaluate their safety profiles and satisfy biomedical regulations.

With an eye on the horizon, a combination of MIP-based DDSs and implantable microchips has great potential for self-regulated drug delivery [5] as the novel DDS can detect changes in the level of a biochemical substance (e.g., glucose) and can prompt the rapid release of the drug (e.g., insulin) under the desired conditions [3]. Striegler proposed a MIP-based α-glucose-biosensor in which pH changes in response to changes in the glucose concentration in the environment [152]. In addition, several interesting studies have achieved controlled drug delivery based on implantable microchips [153,154,155]. Therefore, the hybrid of the MIP and implantable microchips may prove to be a promising drug delivery avenue within reach of clinical applications in the coming years [5,156].

## Figures and Tables

**Table 1:** Specifications of nicotine patch formulations [44,76].

Type of Nicotine Formulation	Nicotine Content (mg)	Surface Area (cm^2^)	Application Duration (h)	In vitro Release Amount
Nicopatch^®^	17.5	10.0	24	700 µg cm^−2^
Nicorette^®^	8.3	10.0	16	500 µg cm^−2^
Nicotine MIP	10.0	1.8	48	2200 µg cm^−2^

**Table 2:** Comparison of different insulin treatments [66,101,103].

Treatments	Administration Route	Loading Capacity (%)	Dosage (IU/kg)	Bioavailability (%)
CS-gold	Oral	53	50	<1
DEX-*b*-PLGA	Oral	25	100	~10
MIP	Oral	82	50	~2
Insulin solution	Oral	-	50	~0
Insulin solution	Subcutaneous	-	5	~100
